# Comparison of Modified Mallampati Score and Ultrasonographic Airway Assessment in Predicting Ease of Glottic Visualization: A Prospective Diagnostic Accuracy Study at a Tertiary Care Hospital in Pakistan

**DOI:** 10.7759/cureus.111072

**Published:** 2026-06-18

**Authors:** M Asghar Ali, Faisal Iqbal, Aiman Shah, Bushra Salim

**Affiliations:** 1 Anaesthesiology, Aga Khan University, Karachi, PAK

**Keywords:** airway management, intratracheal, intubation, laryngoscopy, mallampati score, ultrasonography

## Abstract

Introduction: The accuracy of preoperative airway assessment remains a challenging issue. The modified Mallampati (MP) scoring system has been commonly employed, though with poor predictive accuracy. The ultrasonographic measurement of skin-to-epiglottic distance (SED) has been proposed as an objective tool. We evaluated the accuracy of MP scoring and ultrasonographic measurement of SED in predicting easy glottic visualization based on Cormack-Lehane grading during direct laryngoscopy.

Methods: This prospective observational diagnostic accuracy study involved 400 adult patients who underwent elective surgery under general anesthesia with an American Society of Anesthesiologists (ASA) grade of I and II. Preoperative MP score and SED by ultrasound examination were performed. The degree of glottic visualization was graded according to the Cormack-Lehane scale. Easy direct laryngoscopy was categorized as a Cormack-Lehane scale of I and II, while difficult direct laryngoscopy was categorized as a Cormack-Lehane scale of III and IV.

Results: A significant association was observed between SED and Cormack-Lehane (CL) grade (p < 0.001). The mean SED increased progressively with worsening CL grade (p < 0.001). The area under the ROC curve (AUC) for SED was 0.98 compared with 0.50 for the MP score. At a cutoff >19.5 mm, SED demonstrated 100% sensitivity and 89.95% specificity. The MP score (>3.5) showed sensitivity of 6.25% and specificity of 99.46%. On multivariable analysis, SED was the only independent predictor of difficult laryngoscopy (OR 11.29, 95% CI 4.24-30.07; p < 0.001).

Conclusion: Ultrasonographic SED is a highly accurate and independent predictor of difficult glottic visualization and outperforms the modified Mallampati score. Incorporating airway ultrasound into routine preoperative assessments may improve the prediction of difficult glottic visualization.

## Introduction

Inadequate airway management is among the leading reasons behind patient deaths and morbidity during anesthesia. Hence, it is vital to have a tool that can reliably predict a difficult airway [1]. Several bedside airway assessment tests are available [2], but they exhibit high inter-observer variability and moderate-to-fair sensitivity and specificity. As these tests are subjective, they cannot be used in emergency and critical care settings, as the patient may be confused and uncooperative and may not be able to follow directions [3].

The Mallampati scoring system is often applied when seated to predict a difficult airway and reduce the risk of unexpected consequences associated with difficult airway management. However, the effectiveness of the test is questionable, and it shows a lack of prudence in predicting a difficult airway, as indicated by the number of false predictions [4].

In addition, sublingual ultrasonography has been used for airway management. Recently, it was established that the ability to visualize the hyoid bone with sublingual ultrasonography and the skin-to-epiglottis distance is an objective way of predicting the laryngoscopic view [5-7]. This study aimed to compare the diagnostic performance of the modified Mallampati (MP) score and ultrasonographic skin-to-epiglottis distance (SED) in predicting the ease of glottic visualization based on Cormack-Lehane grading during direct laryngoscopy.

## Materials and methods

After institutional ethical committee approval (ERC number 2024-9086-30934), this study was conducted as a prospective observational diagnostic accuracy study from February 16, 2024, to August 27, 2025, in the preoperative holding areas and operating rooms of the Aga Khan University Hospital (AKUH). Adult patients aged between 18 and 60 years, belonging to ASA physical status I or II, and scheduled for elective surgical procedures requiring general anesthesia with endotracheal intubation were included. Patients were excluded if they had limited mouth opening, maxillofacial anomalies, uncooperative behavior (such as those who were mentally challenged), the need for rapid sequence intubation, a body mass index (BMI) above 35 kg/m², visible skin infection over the neck, a tracheostomy, or the inability to understand either English or Urdu. A non‑probability convenience sampling technique was employed.

The sample size was calculated based on a previously reported incidence of difficult laryngoscopy of 22.7% [8], with an assumed sensitivity of 64.71% and specificity of 78.45%. Using a 95% confidence level and accounting for a 5% potential data loss, the required sample size was estimated to be approximately 400 participants. The calculation was performed using RStudio software (version 4.1.2; RStudio, Boston, MA, USA).

This study was approved by the Ethical Review Committee of our institute (ERC 2024-9086-30934), and informed consent was taken from all the eligible patients. The preoperative airway assessment was done in the clinic by the anesthesia team, and the results were documented in the case records as well as on the pro forma designed especially for the study. On the day of the surgery, the primary investigator personally performed the preoperative airway assessment, and the modified Mallampati score and ultrasonographic airway evaluation results were documented. The ultrasound findings were not disclosed to the primary anesthesiologist performing the endotracheal intubation.

The modified Mallampati score was recorded by asking the patient to sit, open their mouth fully, and protrude the tongue maximally without phonation. The patients were classified into four groups based on the structures visible, i.e., Class I: soft palate, uvula, and tonsillar pillars; Class II: soft palate and uvula visible; Class III: soft palate and uvula base are visible; Class IV: only hard palate visualized [9].

For the ultrasonographic evaluation, the high-frequency linear probe (4-13 MHz) of the Mindray TE7 device was used. During the examination, all participants were placed in a supine position, and their heads and necks were held in a neutral position. The ultrasonographic device was placed transversely over the location of the thyrohyoid membrane, with minimal pressure, and the distance from the skin to the epiglottis was measured in the midline, at end expiration. Three readings were taken, and the average was calculated. To avoid interobserver variability, the ultrasonography was performed only by the primary investigator.

In the operating room, standard ASA monitoring was applied, and patients underwent three minutes of preoxygenation with 100% oxygen via face mask. Anesthesia was induced with intravenous propofol (2 mg/kg) and cisatracurium (0.15 mg/kg). Analgesic choice was left to the discretion of the attending anesthesiologist. After three minutes of bag‑mask ventilation using a mixture of oxygen and air (50%) along with isoflurane (1%), direct laryngoscopy was carried out using a Macintosh blade by an anesthesiologist with a minimum of two years of clinical experience. The laryngoscopic view was documented according to the Cormack-Lehane classification, where grade I represented a full view of the glottis, grade II a partial view, grade III visibility of the epiglottis only, and grade IV the inability to visualize either the epiglottis or glottis [10]. Duration of intubation, number of attempts, and the observed laryngoscopic grade were documented immediately after intubation. Classes I and II, according to the Mallampati test, were taken as predictors of easy intubation and glottic visualization; on the other hand, classes III and IV denoted possible difficulties. Likewise, the Cormack-Lehane grades I & II constituted easy glottic visualization; on the other hand, grades III & IV indicated difficult ones. In instances when patients failed to get intubated for the second time, the protocol for difficult airways was followed.

The data were analyzed using the IBM Corp. Released 2016. IBM SPSS Statistics for Windows, Version 22. Armonk, NY: IBM Corp. The Shapiro-Wilk normality test was performed to check the normality of quantitative variables, which include age, weight, height, BMI, and intubation duration. Data that are normally distributed, i.e., quantitative variables, are represented as mean ± standard deviation, while data that are not normally distributed, i.e., quantitative variables, are represented as median and interquartile range. Categorical variables, which include gender, Mallampati score, Cormack-Lehane grade, and number of intubation attempts, are represented as frequency, percentage, etc. To determine the predictors of difficult laryngoscopy, univariate and multivariate logistic regression analyses were performed. Sensitivity, specificity, and area under the ROC curve were calculated to check the diagnostic accuracy of the Mallampati score and ultrasonographic SED. A p‑value less than 0.05 was considered statistically significant.

## Results

A total of 400 study participants were included in this study (Table 1). Regarding Mallampati score distribution, the majority of patients were classified as Mallampati score II (195; 48.8%), followed by score I (165; 41.3%), score III (36; 9.0%), and score IV (4; 1.0%). Among patients with a Mallampati score I, most were classified as CL grade I (140; 84.8%), while smaller proportions had CL grade II (13; 7.9%), grade III (4; 2.4%), and grade IV (8; 4.8%). For Mallampati score II, 129 (66.2%) patients had CL grade I, 48 (24.6%) had grade II, 8 (4.1%) had grade III, and 10 (5.1%) had grade IV. Among Mallampati score III patients, 15 (41.7%) were CL grade I and 21 (58.3%) were grade II, with no cases in grades III or IV. For Mallampati score IV, 1 (25%) patient was classified as CL grade I and II, while 2 (50%) were classified as CL grade III. No patients with a Mallampati score IV were classified as CL grade IV (Table 2).

**Table 1:** Demographic data BMI: Body mass index, SED: Skin-to-epiglottic distance *Mann Whitney test and chi-square/fisher exact test

Total (n=400)		Easy group (n=368) CL I+II	Difficult group(n=32) CL III+IV	p-value*
Age (years)	Median (IQR)	39 (19)	42.50 (22)	0.648
BMI (kg/m2)	Median (IQR)	26 (6.50)	33.85 (8.68)	<0.001
Gender	MALE	188 (91.7%)	17 (8.3%)	0.825
	FEMALE	180 (92.3%)	15 (7.7%)	
Mallampati Score	I	153 (92.7%)	12 (7.3%)	0.004
	II	177 (90.8%)	18 (9.2%)	
	III	36 (100%)	0 (0%)	
	IV	2 (50%)	2 (50%)	
SED mm	mean +SD	18 (2)	22 (1)	<0.001

**Table 2:** Agreement between Cormack–Lehane (CL) grade and Mallampati (MP) score *Chi-square test/Fisher exact test

Mallampati Score	CL grade, No. of patients (%)				TOTAL	p-value*
	I	II	III	IV		
1	140 (84.8%)	13 (7.9%)	4 (2.4%)	8 (4.8%)	165 (100%)	<0.001
2	129 (66.2%)	48 (24.6%)	8 (4.1%)	10 (5.1%)	195 (100%)	
3	15 (41.7%)	21 (58.3%)	0 (0%)	0 (0%)	36 (100%)	
4	1 (25%)	1 (25%)	2 (50%)	0 (%)	4 (100%)	

Distribution of ultrasound-based measurement (skin-epiglottic distance, SED) according to CL grade showed a progressive increase in mean SED values with increasing CL grade (Table 3). The mean SED for CL grade I (n = 285) was 17.12 ± 1.17 mm. For CL grade II (n = 83), the mean SED was 18.94 ± 1.23 mm. For CL grade III (n = 14), the mean SED was 20.71 ± 0.73 mm, while for CL grade IV (n = 18), it was 22.17 ± 0.62 mm. The overall mean SED was 17.86 ± 1.76 mm. Pairwise comparisons between all CL grades (grade I vs. II, I vs. III, I vs. IV, II vs. III, II vs. IV, and III vs IV) revealed statistically significant differences (all p < 0.001), indicating that SED increased significantly with worsening laryngoscopic grade.

**Table 3:** Distribution of US-based measurement (SED) according to Cormack–Lehane (CL) grade *: Statistically significant (Mann-Whitney U test) p1: p-value between grade 1 and grade 2, p2: p-value between grade 1 and grade 3, p3: p-value between grade 1 and grade 4, p4: p-value between grade 2 and grade 3, p5: p-value between grade 2 and grade 4, p6: p-value between grade 3 and grade 4. CL: Cormack Lehane, SED: skin epiglottic distance

CL grade	No	SED mm		P1	P2	P3	P4	P5	P6
		Mean	SD						
I	285	17.12	1.17	<0.001*	<0.001*	<0.001*	<0.001*	<0.001*	<0.001*
II	83	18.94	1.23						
III	14	20.71	0.73						
IV	18	22.17	0.62						
Total	400	17.86	1.76	-	-	-	-	-	-

**Figure 1:**
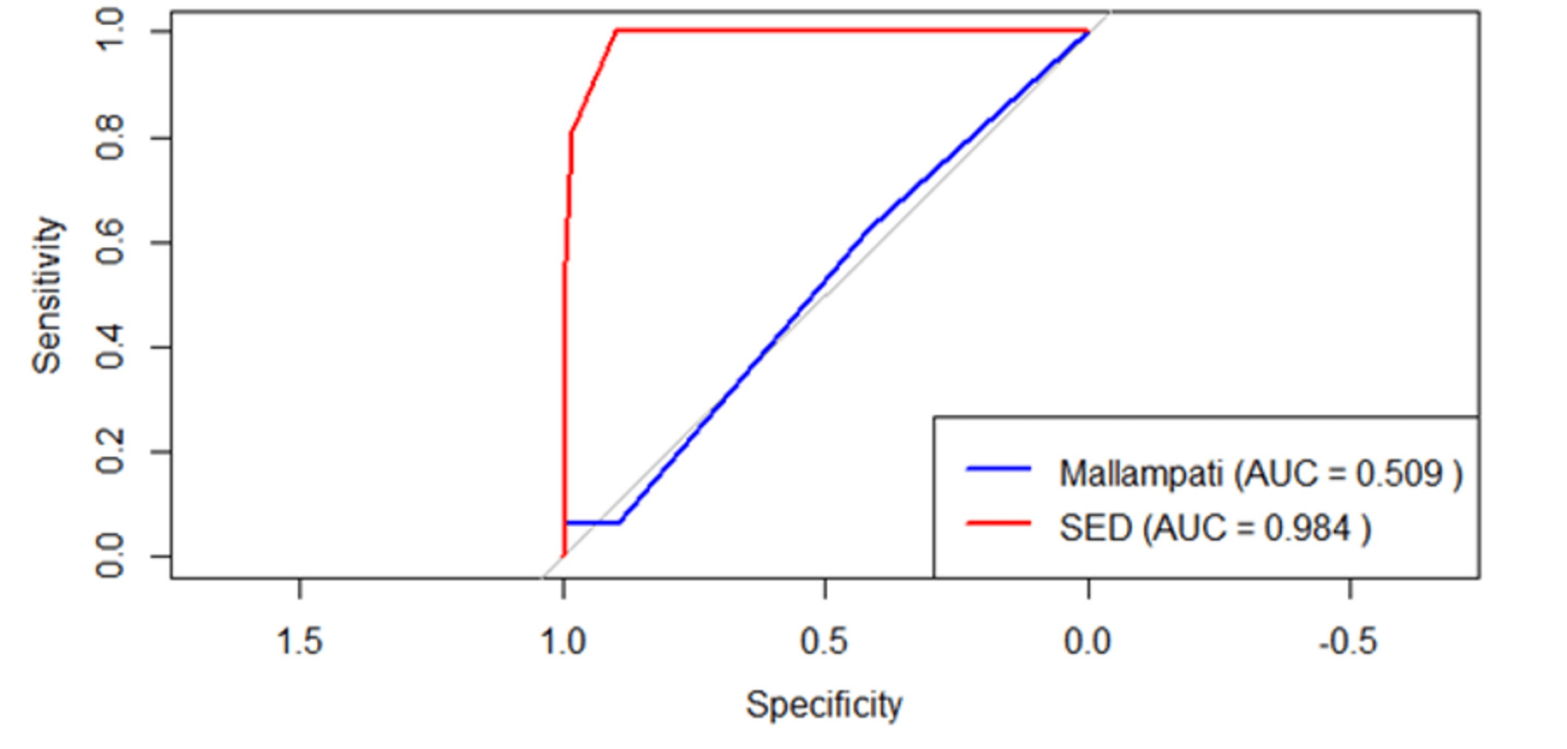
Area under the ROC curve for Mallampati and SED ROC: receiver operating characteristic, SED: skin-to-epiglottic distance

Comparative analysis of the performance attributes of clinical airway evaluation (Mallampati score) and ultrasound airway assessment (SED) in distinguishing easy from difficult laryngoscopy demonstrated marked differences (Table 4). The area under the ROC curve (AUC) for the Mallampati score was 0.50, whereas for SED it was 0.98 (Figure 1). The optimal cutoff value was >3.5 for the Mallampati score and >19.5 mm for SED. For the Mallampati score, sensitivity was 6.25%, specificity was 99.46%, positive predictive value (PPV) was 50.00%, and negative predictive value (NPV) was 92.42%. The p-value was >0.05, indicating no statistically significant predictive performance. In contrast, SED showed a sensitivity of 100%, specificity of 89.95%, PPV of 46.38%, and NPV of 100%, with a statistically significant p-value (<0.05). These findings indicate that SED had excellent discriminative ability compared to the Mallampati score.

**Table 4:** Comparative analysis of the performance attributes of clinical airway evaluation and US airway assessment in distinguishing among cases of easy and difficult laryngoscopy AUC: Area under receiver operating characteristic (ROC) curve, PPV: Positive predictive value, NPV: Negative predictive value, SED: skin-to-epiglottic distance

	MP score	SED
AUC	0.50	0.98
Cutoff value	> 3.5	> 19.5
Sensitivity (%)	6.25	100
Specificity (%)	99.46	89.95
PPV (%)	50.00	46.38
NPV (%)	92.42	100
p-value	>0.05	<0.05

Univariate and multivariate logistic regression analyses were performed to evaluate predictors of difficult laryngoscopy (Table 5). In univariate analysis, BMI (OR = 1.24; 95% CI: 1.15-1.33; p < 0.001), Mallampati score II (OR = 0.07; 95% CI: 0.01-0.60; p = 0.01), Mallampati score III (OR = 0.10; 95% CI: 0.01-0.77; p = 0.02), and SED (OR = 7.85; 95% CI: 4.08-15.11; p < 0.001) were significantly associated with difficult laryngoscopy. Age and gender were not statistically significant predictors. However, in multivariate analysis, only SED remained an independent significant predictor of difficult laryngoscopy (OR = 11.29; 95% CI: 4.24-30.06; p < 0.001). Age, gender, BMI, and Mallampati score were not independently associated with difficult laryngoscopy.

**Table 5:** Comparative analysis of the performance attributes of clinical airway evaluation and US airway assessment in distinguishing between easy and difficult laryngoscopy *Univariable multinomial logistic regression and multivariable multinomial logistic regression BMI: Body mass index, SED: skin-to-epiglottic distance

	Univariate			Multivariate		
	p-value	OR	95% CI	P*	OR	95% CI
Age (years)	0.656	1.05	0.97 – 1.03	0.186	0.95	0.88 – 1.02
Gender	0.825	1.08	0.52 – 2.23	0.585	0.65	0.14 – 3.05
BMI (kg/m2)	< 0.001	1.24	1.15 – 1.33	0.708	1.03	0.87 – 1.22
Mallampati score						
II	0.015	0.07	0.01 – 0.61	0.20	0.01	0 – 11.49
III	0.026	0.10	0.01 – 0.77	0.21	0.01	0 – 12.39
IV	0.997	-	-	0.99	-	-
SED	< 0.001	7.85	4.08 – 15.11	< 0.001	11.29	4.24 – 30.06

## Discussion

In this prospective observational study from a tertiary‑care hospital in Pakistan, we compared the diagnostic accuracy of ultrasonographic skin‑to‑epiglottic distance (SED) and the modified Mallampati (MP) score for predicting difficult glottic visualization. Our results showed that SED was a better predictor of difficult glottic visualization, whereas the MP score was a poor predictor. This is consistent with increasing international and regional literature, which is now strongly advocating airway ultrasound as an objective and reliable method of airway evaluation.

A clear and progressive increase in SED with worsening Cormack-Lehane (CL) grade was observed in our study. Comparable results have been reported in recent South Asian studies. A 2024 Nepalese study showed that the distance between the skin and the epiglottis was significantly greater in patients with difficult glottic views, offering very high sensitivity and specificity (96% and 97.3%) for predicting difficulty [11]. Similarly, an Indian cross‑sectional study revealed that SED was significantly associated with CL grades but found a poor correlation between Mallampati classification and laryngoscopic grade [12]. These findings again highlight the limitations of MP scoring, particularly in settings where there is a high degree of variability in patient cooperation, posture, and examiner skills, factors that are common in the clinical scenario in Pakistan.

International literature also supports the use of SED as a reliable measure for predicting the ease of glottic visualization. A 2025 Brazilian study reported that ultrasound‑measured cutaneous‑epiglottic distance (CED) showed a high area under the ROC curve (AUC 0.90), thus being a good discriminator for identifying difficult laryngoscopy in viewing the glottis [13]. Likewise, a prospective observational study from Türkiye in 2025 found that SED was significantly higher in patients with a difficult laryngoscopic view, and it was a good predictor for a poor laryngoscopic view compared to other conventional methods such as Mallampati [14]. Soltani et al. also reported that the ratio of skin to epiglottis distance and epiglottis to mid-vocal cord distance (DES/EMVC) was significant (p=0.004) for predicting higher Cormack-Lehane grades in patients with BMI>25 [1]. According to Gomes et al., the skin-to-epiglottis distance proved to be a useful predictor of difficult glottic view (p = 0.02); on the other hand, the hyomental distance in the neutral position proved to be the most reliable ultrasound assessment parameter as a preoperative airway assessment tool [15]. According to Carsetti et al., although CED exhibits high accuracy and precision, it is more likely to be most useful in situations where CED yields negative results, predicting an easy laryngoscopic glottic view in about 95%‒97%. This has been attributed to the low prevalence (10%‒20%) of difficult laryngoscopies with higher CL grades in the sample population. A positive test for CED, on the other hand, implies about a 30%‒50% likelihood of the presence of a poor glottic view [6]. 

However, it is worth mentioning that the predictive value of the Mallampati classification was low in our study, as the AUC value was 0.50, and the sensitivity was just 6.25%. The results were similar to those of Chatterjee et al. [16], who stated that the traditional (in sitting position) Mallampati test exhibited low predictive value and did not have a significant correlation with Cormack-Lehane grading, while the supine Mallampati test had higher correlations. Anushaprasath et al. [12] also reported that the sensitivity of the Mallampati score was 60.87% for higher CL grades and had poor positive predictive value (50%).

An interesting finding from our study is that the cutoff point for the SED for our population was calculated to be 19.5 mm using the ROC curve for the determination of the Youden optimal cutoff, with a high predictive accuracy for the diagnosis of difficult glottic visualization (AUC 0.98, 100% sensitivity, 89.95% specificity), compared to the internationally recommended cutoff point of 21 mm. This can be explained by a number of factors. Firstly, the anthropometric variations may have contributed to the different cutoff points for the South Asian population compared to the Western population. Individuals from South Asia tend to have smaller bodies than their Western counterparts, which may explain the use of lower neck circumference cutoffs [17]. Studies in the Indian population have demonstrated relatively lower values >15.5 mm [12], suggesting that ethnic and anthropometric variations may influence airway ultrasound measurements. In another study on Indian parturients, the mean skin-to-epiglottis distance has been reported to be approximately 19.9 mm [18]. On the contrary, higher cutoffs in global research have been attributed to greater baseline tissue thickness; for instance, the Brazilian study established a cutoff for cutaneous epiglottis distance (CED) of 25.6 mm [13]. The study carried out in Turkey also recorded increased SED values in challenging airways due to anatomical differences [14].

These findings have considerable implications for the management of the airway in the Pakistani population. Ultrasound assessment of the airway has several advantages over other bedside assessment tools. It is non-invasive, objective, and requires minimal patient cooperation. This is particularly relevant for the Pakistani healthcare system, where the accuracy of other bedside assessment tools may be compromised by the constraints of the healthcare system. The increasing availability of ultrasound machines in both public and private healthcare facilities would further facilitate the use of this tool for the assessment of the airway.

A significant strength of this study lies in the large sample size, which comprises 400 patients, making this study highly precise and reliable in terms of estimates, as compared to other previous literature on airway ultrasound. The use of a standard ultrasound technique, where all the ultrasound procedures were conducted by a single investigator, also adds a significant strength to this research, as a high degree of consistency was maintained in the study. Moreover, anesthesiologists conducting laryngoscopies, with a minimum of two years of experience, also eliminate the factor of operator skills, which could have affected the grading of laryngoscopies. This study is also significant, as it is one of the first studies conducted on a Pakistani population, compared to other existing literature on SED, which makes this research highly reliable in terms of estimates. However, certain limitations need to be addressed. First, this was a study done in a single center, a major tertiary care center, which might restrict the applicability of the results to smaller centers and even resource-constrained settings in Pakistan. Second, although the use of a single operator for the ultrasound examination helped eliminate inter-observer variability, it also meant that the reproducibility of the SED scores between different clinicians could not be assessed, which is an important factor for widespread applicability. Third, all laryngoscopies were performed by relatively experienced anesthesiologists, and the results might not hold for settings where trainee doctors or less experienced clinicians perform the procedure. Fourth, the exclusion of patients with a BMI > 35 kg/m² might restrict the applicability of our results for obese patients, even though the incidence of obesity is on the rise in this region. Furthermore, the study did not assess emergency airway situations, trauma victims, or patients with diminished levels of cooperation, which are also important patient groups in which airway ultrasound might be particularly useful. Yet another limitation of this study is that the rate of difficult glottic visualization in the study sample was comparatively low. As difficult glottic visualization happens to very few patients, the lower rate of events might have compromised the accuracy of our estimates. Finally, though the MP score was directly compared with SED, other important predictors, such as thyromental distance, hyomental distance, and neck circumferences, were not included, which might have given further context or improved multivariable modeling.

The findings suggest that SED may be associated with poor visualization of the glottic opening and could serve as a useful adjunct in preoperative airway assessment. Incorporating SED into routine evaluation may help anesthesiologists identify patients at increased risk more effectively; however, further prospective and multicenter studies are needed to validate its predictive utility and comparative performance. By establishing the cutoff value of ultrasonographic SED at 19.5 mm, the current research has provided a clinically relevant tool that is specific to the Pakistani population, which may be of benefit in the prediction of difficult glottic visualization and the overall improvement of patient safety by the widespread adoption of airway ultrasound in the field of anesthesia in Pakistan.

## Conclusions

Ultrasonographic skin-to-epiglottic distance demonstrated better diagnostic performance than the modified Mallampati score for predicting difficult glottic visualization in this single-center cohort of elective ASA I-II adult surgical patients. However, the proposed cutoff requires external validation, and the findings should be interpreted cautiously given the convenience sampling design and limited number of difficult laryngoscopy cases.
